# Implementation of Automated Guided Vehicles for the Automation of Selected Processes and Elimination of Collisions between Handling Equipment and Humans in the Warehouse

**DOI:** 10.3390/s24031029

**Published:** 2024-02-05

**Authors:** Iveta Kubasakova, Jaroslava Kubanova, Dominik Benco, Dominika Kadlecová

**Affiliations:** Department of Road and Urban Transport, Faculty of Operation and Economics of Transport and Communication, University of Zilina, Univerzitna 8215/1, 01026 Zilina, Slovakia; iveta.kubasakova@uniza.sk (I.K.); dominik.benco@stud.uniza.sk (D.B.); kadlecovadominika502@gmail.com (D.K.)

**Keywords:** AGV, AMR, automation, optimization, analysis

## Abstract

This article deals with the implementation of automated guided vehicles (AGVs) in a selected company. The aim is to analyse the use of AGVs in our country and abroad and to provide information about the use of AGVs in other countries and operations other than ours. The result of the analysis was a literature review, which points out the individual advantages and disadvantages of the use of AGVs in companies. Within the review we also address the issue of AMR vehicles, due to the modernization of existing AGVs in the company, or the replacement of AMRs with AGVs in general. Our aim is to show why AGVs can replace human work. This is mainly because of the continuous increase in the wages of employees, because of safety, but also because of the modernization of the selected company. The company has positive experience of AGVs in other sites. We wanted to point out a higher form of automation, and how it would be possible to use AMR vehicles for the same work as AGVs. In the company, we have identified jobs where we would like to introduce AGVs or AMR vehicles. Consequently, we chose the AGV from CEIT operated by magnetic tape and the AMR from SEER as an example. Based on studies, the demand for AGVs is expected to increase by up to 17% in 2019–2024. Therefore, the company is looking into the issue of the implementation of AGVs at multiple sites. The question which remains is the economic return and the possibility of investing in the automation of processes in the company, which we discuss in more detail in the conclusion of the article and in the research. The article describes the exact processes for AGVs, their workload, and also the routes for AGVs, such as loading/unloading points, stopping points, checkpoints, junctions with other AGVs, charging stations, and field elements, as well as their speed, frequency and the possibility of collision with other AGVs. Our research shows that by applying the new technology, the company will save a large amount of money on employee wages. The purchase of two AGVs will cost the company EUR 49,000, while the original technology used in the company cost EUR 79,200 annually. The payback period for such an investment is 8 months. The benefits of implementing AGVs are evaluated in the last section of this paper, where both the economic and time requirements of the different proposals are included. This section also includes recommendations for improving specific parts of the enterprise.

## 1. Introduction

Many companies in Slovakia are implementing AGVs in their warehouses, as one of the conditions of technological innovation within Logistics 4.0. is the implementation of AGV in the warehouse. We can thus eliminate errors in material handling, and we can eliminate safety risks in the event of a collision between material handling equipment and a human being. 

The reason why AGVs are the right choice is that AGVs are flexible and able to rotate 360°. In the company, a small sized AGV is used to transport the load placed on top of the AGV or to transport the trolleys in the towing set (there can be only one). The AGV is constructed using a rigid steel structure which allows a fixed structure to be mounted on top. Because of this possibility, the AGV can be turned into a workstation in the assembly line. This type can also move by suddenly turning and thus change direction 360° around its own axis. Furthermore, other active systems can optionally be added as required, e.g., a roller conveyor or a lifting platform. The remaining parameters of the AGV can be found in Table 1 below.

**Figure 1:**
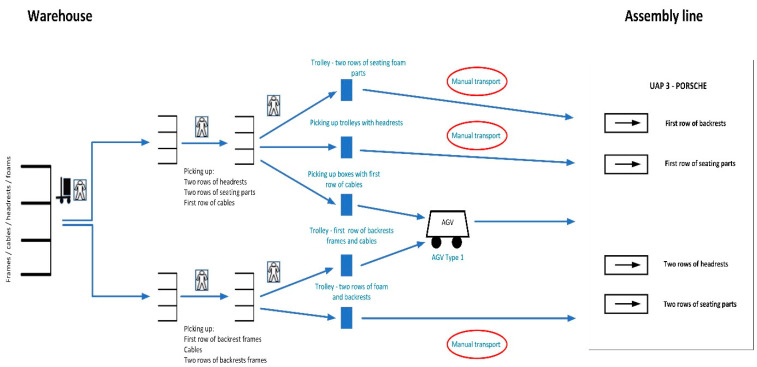
Schematic diagram of material transport from to the warehouse to the assembly line.

The entire picking up process starts from the moving of the goods into the warehouse from the truck, where a worker uses a forklift to unload the frames, headrests, and foams (backrests and seating sections). Two other workers then place the goods in the warehouse and pick up the material for the specific line from the trolleys. These workers distribute the material they are picking up. The goods are then transported with the help of other workers to the production line. So, the four operators take turns with each other to carry out the given work activities. The workers transport the goods using three types of trolleys and one AGV. The assembly line is composed of four stations, to which different kinds of transport lead. At the assembly line, the material trolleys are unloaded and at the same time empty trolleys are picked up and transported back to the warehouse. This process is repeated throughout the operation. The diagram is shown in Figure 1. 

Automation took place on the trolley routes where manual transport was carried out using hand-pulled trolleys and operators, which are indicated in the red circles.

### Basic Information on the Material Transported 

The focus is on a part of the production hall only; this part focuses on the production of seats. Seats are made in three colour variants (black, red and beige). 

Weight data of transported materials:

- Back foam, approximately 2.2 kg;
- Foam intended for seating; this may vary depending on where the foam will be placed:
  ○ First row of seats of approximately 1.6 kg;
  ○ Second row: 40% of approximately 1.5 kg;
  ○ Second row: 60% of approximately 2.4 kg.
- Head restraints of approximately 0.7 kg.

The vehicle’s capabilities are pre-set to monitor the route ahead, accounting for people, other machinery and other AGVs. There are other types of AGVs operating in society that can only move in a two-way operation (back and forth), with no turning of these devices. Therefore, there is a requirement for necessary communication afterward with other types of AGVs to avoid collision. A safety system must be built into the AGV to perform this task, by means of which order and intersection control is performed. There are also collisions between employees and AGVs in the workplace, which evokes a further requirement in terms of upgrading the system in terms of safety. For this, the AGV uses sensors, and as soon as a “pre-obstacle” comes into its path the AGV automatically stops its operation and waits for its removal. There is a scanner embedded in the system which is equipped with four safety zones that can be configured in its superimposed circuit by means of RFID tags. Furthermore, it can be connected to control systems, which have an even higher level of maintenance, and process systems. The AGV has an integrated display with diagnostics on the front. The display informs people about the status of the device, possible warnings, battery status, and its operation target. The AGV can also be operated without the aid of a central control, using a simple operator panel located next to the information display. The AGV can operate continuously, providing complete autonomy 24 h a day. This capability is made possible by two 200 W batteries, with a 90 Ah battery life. It incorporates a differential drive unit which is used to carry out specified tasks in conjunction with the tilting system. The type of AGV used can move on a pre-prepared infrastructure, in which magnetic guidance needs to be incorporated and an integrated charging system, located in a loop, needs to be built into the floor.

The installation can only be implemented indoors or outdoors, in which case it must be covered because the vehicle cannot operate in rain, fog or direct sunlight. In terms of surface maintenance, the surface on which the AGV runs along its route must be in good condition, smooth, clean, free of damaged parts, and must not be made of slippery or metallic surfaces.

## 2. Literature Review

In terms of the current and recent world situation, the period of the COVID-19 pandemic has played a significant part in the shift towards automation. This has had a major impact on world trade levels, which have been disrupted by a shortfall in human labour. In this context, the demand for alternative storage systems has suddenly increased. This led to a search for ways to resolve the “outages”, which had to be minimized. One of the main solutions that increases the efficiency of labour productivity in a company is automation. Based on the period mentioned above, it is significant that companies have started to introduce different types of AGVs in most of the manufacturing, warehousing, packaging stations [1] and various other parts of the companies. AVGs not only minimize the transportation time of goods but also minimize the cost of company operation, to a considerable extent. If we look at automation from a foreign perspective, specifically in China, JD.com was a pioneer in business-to-consumer e-commerce in 2010. The company has built integrated warehouses to maintain its market position, address the urgent need to meet growing demand and maintain high quality logistics services. The mentioned technology has helped the company to reduce its cost-to-fill ratio to a world-leading 6.5%. Other benefits have led to savings of hundreds of millions of dollars. During 2020, the company delivered its retail orders on the same day or the next day at the latest. Meeting the first- or next-day delivery criterion accounted for up to 90% of deliveries [2].

The field of AGV design research is not exhausted, as new applications and products are constantly emerging. Despite the increasing investment in developing smarter businesses and improving manufacturing competitiveness, AGV R&D in important areas such as SMEs is still lacking. So the problem of AGV position is still open. It can be defined as a control system that seeks a control input that minimizes the measured error compared to a trajectory used as a reference, considering the operational requirements and constraints of the vehicle and the environment. The first step of AGV operation is the correct path. Accurate positioning is a key factor in AGV control. Increasing AGV positioning accuracy improves the accuracy of AGV route tracking and localization, reduces the possibility of accidents during operation, and reduces the cost of building complicated infrastructure [3].

The manufacturing environment is steadily increasing the impact of systems which offer the possibility of dynamic adjustment to the AGV [4]. Dynamic AGV planning is based on a “digital twin” system. The digital twin is built in a virtual space in which a framework for real-time interchange and real-time control based on dynamic data is established. It consists of real-time data exchange and dynamic task-list generation, determining the number and speed of AGVs in different working conditions and effectively improving the efficiency of the logistics system. The traditional logistics operation planning strategy is unable to meet the requirements that are currently focused on higher transportation efficiency [5].

The control of the position of the lower level AGV is based on a well-defined guide. Such guides include a coloured strip or magnetic tape attached to the ground, or even virtual paths. Virtual paths are not physical. They can be planned based on environmental mapping, for example, using laser sensors, or based on physical tags (such as RFID, QR code tags, or laser reflectors) that do not reveal the planned trajectory. The purpose of the control is to keep the vehicle on the trajectory and meet the requirements of the control theory, such as steady-state error, overshoot value, and adaptation time. One of the navigation modules is the vehicle motion control. Hybrid architectures also combine higher- and lower-level controls operating at different time intervals and different targets. Thus, navigation techniques and lower-level control are not mutually exclusive. Hence, this paper considers navigation techniques as a higher level of deliberative control [6].

Automated guided vehicles in the factory, called AGVs, were also developed. Many countries are investing a lot of energy and resources in developing smarter factories and increasing the competitiveness of manufacturing. The AGV is one of the essential pieces of equipment for replacing labour and reducing the delay in the whole production process.

Route planning and AGV movement should be based on image detection of the surrounding environment. Current AGVs are mostly guided by a magnetic line on the floor or a rail on the ceiling, which leads to a significant increase in construction costs if the plant layout is changed. AGVs should learn how to avoid obstacles and move to the target systematically, through image detection and deep learning. Precise positioning is also important for AGV driving. Currently, the position of an AGV is calculated from its velocity and movement time, and its position error is more than a few meters, due to external factors such as the frictional force of the floor or rail [7]. 

Recently, scheduling and operation issues have been considered as some of the most important issues in manufacturing and logistics systems. In particular, transportation planning using automated guided vehicles (AGVs) in steel production, semiconductor manufacturing and warehouse systems has been widely studied from both theoretical and practical perspectives [8]. 

The location and the map are the basis of the journey planning. The AGV located in the local pro centre is implemented based on SLAM, which means simultaneous localization and map generation. Under the condition that no prior information about the best environment is available, the AGV can determine its position in the environment and estimate the location for the best motion using a mounted sensor. In a global situation, its position on the environment map can be determined by obtaining external steady-state information. The SLAM localization model and the characteristics of different maps lay the foundation for AGV path-planning research. Visual SLAM realizes the construction of the environment map using the process of movement by camera, and the specific structure is shown in figure in Section 3.2.1, including the following: sensor information reading (using sensors to read image information and preprocessing); a visual odometer (calculating camera motion and position and obtaining the appearance of the local map through the relationship between neighbouring images); nonlinear optimalization of image processing (processing camera position information and loop-detection information accumulated by the visual odometer at different times and optimizing this information to obtain the motion trace); loop detection (assessing whether the loop was in a previous position); and mapping (creating a map of the environment by estimating the trajectory of the camera motion). The main principle is to use the difference between neighbouring frames to evaluate the camera’s own motion and then obtain information on the local position of the AGV. An efficient ad hoc request-answering method based on an autonomous decentralized method is proposed for the AGV routing problem. First, each AGV as an agent finds the shortest path that satisfies the requests assigned to it. If potential collisions (predicted collisions that will occur if no precautionary action is taken) are detected, one of the two AGVs, as selected based on the bargaining rule, adjusts its route. A set of negotiation rules shall be used for each collision-avoidance action. These rules consist of a condition part and an action part. The rule that corresponds to the conditions of the two agents involved in the following collision is selected from the rule set [9,10].

AGVs with path constraints such as a well-designed path layout (good infrastructure, i.e., a network path structure with short distances and efficiently used paths with little blocking (congestion), and good-sized input and output buffers with minimum waiting times at stations, are very important. Although the routing of a free AGV is more flexible, even for this type of vehicle in most cases a route layout will be defined to limit the number of possible routes, thus facilitating operational control.

The number of AGVs required is the sum of the total loaded- and empty-trip time and waiting time (due to congestion, among other things) of the AGV in the busy time period, divided by the time an AGV is available during that period. Since this is a design problem, the computation can be rough, e.g., in [6], no waiting time is considered. Preventing AGV collision is a common problem. Current solutions such as inertial and laser guidance have low flexibility and high environmental requirements. Other disadvantages are, for example, hardware requirements, poor flexibility, and difficulties with complex working environments [11]. 

In a traffic system with multiple automated guided vehicles, AGV-path conflicts directly affect the efficiency and coordination of the entire system. At the same time, uncertainty in the number and speed of AGVs will lead to excessive costs. To solve these problems, the AGV Multi-Objective Dynamic Scheduling (AMODS) method, which is based on the digital twin of the workshop, is proposed. The digital twin of the workshop is built in a virtual space, and a real-time bidirectional exchange-and-control framework based on dynamic data is established. The digital twin system is adopted to exchange real-time data, create an updated real-time dynamic task list, determine the number of AGVs and the speed of AGVs in different working conditions, and effectively improve the efficiency of the logistics system. The architecture of the dynamic-scheduling workshop logistics system based on the digital twin is composed of the physical world, the virtual world, the digital twin, and the service management system [12]. 

To improve the safety and flexibility of AGVs, a collision-avoidance system based on INS (Inertial Navigation System)-UWB (ultra-wideband) is being introduced. Compared with traditional inertial and laser guidance systems, the proposed method is more flexible and cheaper, with good accuracy and stability. For accurate positioning, an electronic warehouse map is created where the UWB anchor nodes are deployed. The localization coordinate of the AGV is obtained by three nearby UWB anchor nodes on the map. To improve the positioning accuracy, the EKF method, which integrates INS and UWB data, is used. To avoid collisions, the current position of the AGV and its motion data are used to predict its next position, to reduce the effect of AGV steering delay. Experimental results from other studies show that the proposed method achieves accurate position estimation and that the AGV effectively detects obstacles and avoids potential collisions. The positioning technology is an important technological feature of the AGV [13,14]. 

The world’s advanced organizations also use machine determination to select strategies for planning AGV technology in production. Among such technologies, we can include one timeless technology we call artificial intelligence. The development of intelligent manufacturing environments, which include AGVs, will drive the future. In this case, AGVs play an important role in manufacturing systems, as they have the potential to improve internal logistics. Improvements in internal logistics are about increasing production flexibility and, consequently, the productivity of the whole system. However, this depends on a good-quality schedule, which, if properly set up, can be used to minimize production costs and minimize the overall production time [15].

To achieve the maximum satisfaction with the technology, feedback from the devices already in place needs to be monitored. AGVs need also to be monitored and checked to ensure that they are performing correctly and adhering strictly to their trajectory. As many companies have implemented AGVs, they form an integral part of their logistics processes. When performing dynamic activities, a so-called “estimator” is needed, where, for example, the ensemble Kalman filter algorithm can be used. This algorithm consists of a simulation in which states are observed. These states are composed of two activities:

- Observation of the speed;
- AGV system diversion rate [16].

Before implementing AGVs in the company, it is necessary to consider all important aspects concerning the layout of the production part of the company, the location of the warehouse, the handling aisles, the parameters of the future goods to be transported, and whether it will be appropriate to introduce AGVs in the company in general. The actual selection of AGVs and the eventual redevelopment of the hall in favour of the use of AGVs will depend on this. The return on the equipment in question, if it is set up correctly, is always almost instantaneous, whether in terms of time or money, compared to manpower. Looking at the system from the point of view of the actual planning of the introduction of AGVs into the enterprise, it is necessary to specify the planning activities, which consist of the following:

- Task scheduling;
- Path planning;
- Traffic control management.

These activities are essential, as they are the basis for the optimization of travel times associated with the minimization of system costs. In a proper scheduling solution, the shortest route is assigned to the AGV. Achieving conflict-free scheduling of AGVs, with multiple cargo types, represents a challenging process in production logistics and transportation [17].

The continuous development of production systems in the field of internal logistics has brought new challenges, for which innovative automation solutions have been developed [18]. Innovative solutions are also emerging due to growing material supply chains with the requirement for rapidly changing logistics structures. These require flexible material flow solutions, and these in turn increase the use of autonomous mobile robots [19]. AMRs are used in developed organizations that are constantly moving forward with the trends in development. Industry 4.0 includes innovative technologies that include autonomous mobile robots and collaborative robots. These robots are specified to have higher cost-effectiveness and flexibility. Hence, they are more suitable for automated internal logistics systems in a company [20].

Nowadays, autonomous mobile robots are the latest to be introduced into enterprises instead of AGVs, due to their practical use in the “modern” world of logistics [21]. AMRs and AGVs are suitable for deployment in hazardous parts of work areas [22], where there is a high probability of worker accidents. In this case, for example, the handling of material of too large dimensions or with too high a weight may be involved. During implementation, the company determines which auxiliary elements the automated system can work with. For example, lasers can be used to sense the necessary information, or radio frequency identification (RFID) technology can be applied in a logistics information system. The technology can also be used in transport and warehousing. The introduction of RFID significantly increases the efficiency of logistics processes [23].

With the increasing competition in the automotive industry, technical developments in various sectors are also increasing. This impact makes it imperative for manufacturing logistics companies to improve production efficiency while reducing costs and increasing economic benefits to the society, at the same time. Flexible automation clearly contributes to this [24]. Automation is increasingly coming to the fore in meeting these objectives. The adoption of this technology increases flexibility, accuracy, productivity, and rapid return on investment in its implementation. However, companies often ask questions related to the implementation of the above-mentioned devices [25].

Today’s smart manufacturing facilities are becoming more modular day by day and must be constantly reconfigured, as this is required by logistics processes [26].

AGVs belong to the Industrial Revolution 4.0 (Industry 4.0) [27]. The industrial revolution mentioned above is being pushed forward by the Industrial Internet of Things and physical computational systems; these are creating intelligent information technologies that are transforming supply chain analysis, monitoring, and automation [28].

Because AGVs have become an essential part of flexible manufacturing systems, automated data aggregation in the Industrial Revolution 4.0. is very beneficial from the data clustering point of view [29]. The Fourth Industrial Revolution can be classified as very helpful in the COVID-19 era, where this Industry 4.0 has made the work in factories easier and simpler when dealing with employees. Failure to adopt this technology can leave the company behind in operations [30]. 

According to these authors, simulation models provide a more detailed and understandable representation of order-picking processes and operations compared to analytical models. This is a very important issue for warehouse managers, who can use simulation models as decision support tools to design efficient logistics facilities such as warehouses, or to improve existing facilities of this kind in the real world (by analysing the interaction between operations and processes in simulation mode, without any risk from such analyses in real facilities). The model presented in the paper is particularly applicable to industries that deal with the handling of multiple small units, especially in selected major industries such as the electronics industry, food industry, textile industry, automotive industry, chemical industry (pharmaceutical industry), steel industry and construction industry [31,32].

Automated guided vehicle systems are used for tasks that would normally be performed by forklifts, conveyor systems, or hand trucks, which move large volumes of material repeatedly [33]. 

In addition to transporting raw materials, AGVs are used in work-in-process applications and with finished products to support production or production lines. In work-in-process applications, AGVs move materials or parts from warehouses to production lines or from one workstation to another, ensuring the repetitive efficient movement of materials during the production process. Without AGVs, production processes can come to a halt when the work-in-process lines run out of material. Production is then delayed while a human worker picks up the necessary materials from the warehouse and delivers them to the production line. 

AGVs are also used in infeed and outfeed handling for replenishment and for picking up. For example, AGVs can be used to transport inventory from the point of receipt to the point of storage, or from long-term storage locations to picking-up locations for replenishment. Moving inventory from long-term storage to picking-up locations ensures that the people picking up the goods will have adequate inventory, thereby streamlining the picking-up process. AGVs assist in the picking-up process by guiding warehouse co-workers through tasks and transporting picked-up orders to packing and fulfilment sites [34]. 

## 3. Materials and Methods

### 3.1. Technological Procedure after the Introduction of AGVs

The introduction of AGVs has eliminated the number of staff who have been redeployed to other necessary positions. In the process, 3 manual workers who were designated to transport material from the warehouse to the production line were eliminated. The workers also loaded the respective trolleys with materials and picked up materials independently, as needed. Automation was carried out on the remaining transport from the warehouse to the assembly lines and back.

In the replacement of 3 workers, the company has deployed 2 AGVs. For AGV workflow-loading and -unloading points, 3 unloading points are needed for the AGVs on the line:

- The 1st place is for a trolley with foam backrest reinforcements;
- The 2nd space is designated for a trolley with foam seat cushions;
- The 3rd space is reserved for a trolley with head restraints.

**Figure 2:**
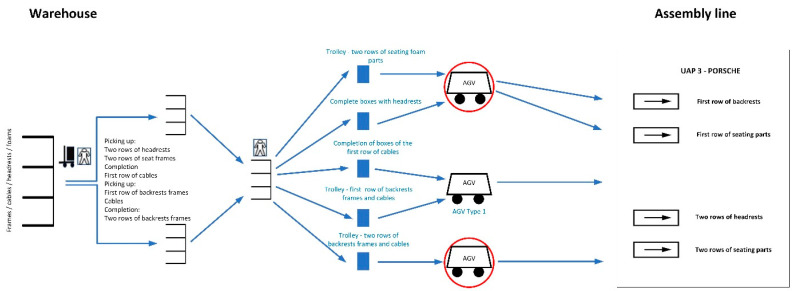
Automation at the selected location.

Every single place is located at different parts of the line. In terms of loading, the unloaded trolleys are placed by the AGV at one designated location in the warehouse. There, one worker, who selects all 3 types of materials for the respective trolleys, takes the trolley to the warehouse, then loads it with new goods and places the loaded trolley at the location (in the warehouse) where the AGV will later take over the loaded trolley (see Figure 2). The AGV can be independently loaded and unloaded using a lifting platform without the need for an employee. When unloading a full cart in the line, the AGV loads the empty cart to eliminate “empty” AGV trips and transports it to the front of the warehouse.

Automation took place on trolley routes where manual transport was carried out (with previous technology) using hand-pulled trolleys and operators. The red circle indicates the locations of deployed AGVs of small dimensions. The automatic charger is built into the floor in a dedicated charging area. The AGV does not need to charge while stationary, as there may be a situation where one AGV will be charging and the other will be waiting for a request behind it (in a loop). The AGV station is controlled by the First In, First Out (FIFO) system. This system uses the conditions for ordering the FIFO system, which are as follows:

- The recovery of the transit signal for the FIFO order is carried out by an RFID tag in the loop, after the AGV leaves the production zone of the line;
- From this point, the AGV starts communicating with the power-line communication (PLC) and announces that the order is complete.

Faurecia, together with the AGV supplier, during the installation process, determined the exact position of this tag. Each material must have its own tag so that the AGV can identify which production line needs to be supplied with the material at that moment.

#### 3.1.1. Control Point

The company that supplied the AGVs prepared one control point which was used to communicate with the AGVs. The position and the subsequent route are checked to see if it is free or not at the required moment, to avoid a situation where the first AGV blocks the second AGV from performing the task. If the situation is different, i.e., an AGV enters the route of another AGV, the first AGV stops and allows the route of the other AGV to be completed.

#### 3.1.2. Traffic Intersection with Other AGVs

AGVs from other suppliers are also in place in the company, and on some routes they cross with the newly introduced AGVs in Segment 2. For that reason, the system needs to be upgraded, and therefore the junctions adapted, to be suitable for all AGVs. The mentioned crossing occurs in the middle of the route (from the warehouse to the production line), where the main handling aisle is located. The communication consists of sending signals between AGVs, so the communication between AGVs must be two-way (between new and old AGVs). If a newly installed AGV wants to enter a common traffic zone, the PLC system checks whether the zone of the previous AGV is occupied or not. If the route is automatically cleared, entry is blocked for older-technology AGVs and allowed for newer-technology AGVs. The new AGVs have three positions for entering and three positions for exiting this zone.

#### 3.1.3. The Charging Station

A charging station is installed inside the loop and is operated as a stop for the AGV. The charging station is installed on a pole in the form of a box. One cable with three wires is installed for each charging point. 

#### 3.1.4. Trolleys 

The compact trolleys for AGVs were designed by Faurecia independently, according to the adaptation of the material to be transported and the increase in the transport capacity of the trolleys.

#### 3.1.5. Elements of the Field 

A PLC1512 in an enclosure with a basic human machine interface (HMI) panel, type KTP 1200, and an industrial switch will be used to control the entire system. We will use 2 Integrated Facilities Management (IFM) units, type AL1102, to collect signals from the assembly zone.

### 3.2. Functions of the Circuit

PLC—this is the brain of the entire AGV system. In this project, a PLC1512 is used, connected via PROFINET to the IFM AL1102 modules and via standard networks to the customer’s Wi-Fi network. The PLC controls all signals in the field and all communication with the AGVs.

Wi-Fi—all AGVs installed in this project use a Wi-Fi network to communicate with the parent system, and the network had to be secured by the company. The AGVs will always communicate with the parent PLC 1512.

HMI—the HMI panel is installed in the main PLC box. The purpose of this panel is to monitor all plant traffic, along with traffic zones and signal monitoring. There are also password-protected functions in the HMI panel so that, in the event of an emergency, traffic, zones, and traffic signals for the AGVs can be restarted. The default language of the system is English, but the Slovak language can be added. The system can be pre-configured, but currently operates on the following principle:

Screen—Intersections: a total of 30 intersections divided into 3 sub-screens. Each screen will display 10 swipe buttons. The button changes colour depending on the status of the junction (e.g., whether it is busy or not). If the advanced user is logged in, clicking the button will re-execute the intersection.

Screen—Signal monitoring: here, all connected inputs are displayed and there is the option to bypass them sequentially.

Screen—Layout: this displays the factory layout, with all positions. If the AGV is present at the stop on the charger, it will be displayed with a green rectangle. Also, for all positions with a sensor, the status will be displayed with a circle (a green circle means that this station is ready for a new job, a red circle means that it is not ready for a new job, and if there is a situation in which all stations are ready to send a job, they will all be shown with a green circle). To perform the check, the visualization is the same. If the AGV can enter the sensor position, it will be shown in green.

Screen—AGV list: in this case, the status of all connected AGVs is displayed.

Screen—Alarm History: here, you will see a list of errors collected from the AGV with the time they occurred, sorted by time interval. The line also contains information about the serial number of the AGV. If there is an error, the line is shown in red; if it is only a warning, the line is shown in orange.

Intersections—the project includes central control of intersections where 30 intersections will be prepared with AGVs that can be monitored from the HMI.

Photocells and inductive sensors—5 inductive sensors are used to monitor positions in the assembly zone and warehouse. Four sensors are used to create the order and one sensor is used to check and block the AGV entrance.

If we look at the logistics results measured by the introduction of AGVs, we can see in Table 2 a summary of the logistics efficiency information, where basic information is summarised over one working shift.

**Figure 3:**
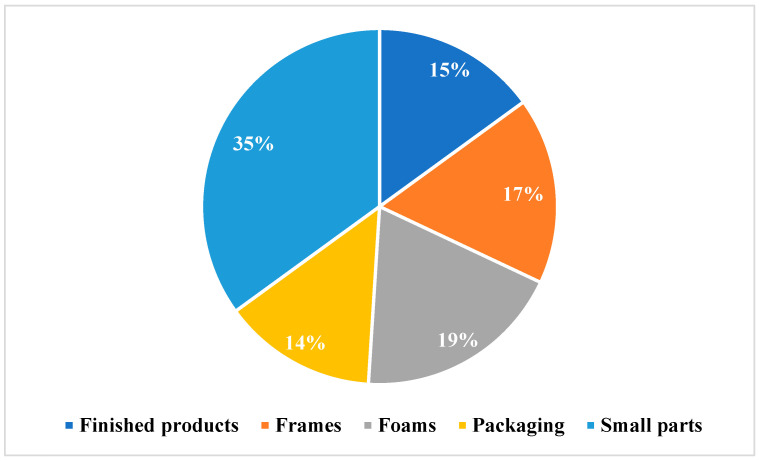
The workload.

Additionally, the workload, which is shown in Figure 3, is divided into several parts (by commodity), frames, foams, packaging, finished products, and small parts—plastics, screws, levers, buttons, etc.

From the graph, we can see that the item with the highest time is the picking up of small parts, with a value of 35%, and, on the contrary, the item with the lowest value is the item specified under the name “packaging”, which accounts for 14%. The remaining commodities show approximately the same percentage of workload as the item packaging.

#### 3.2.1. Proposal for AGV/AMR

**Figure 4:**
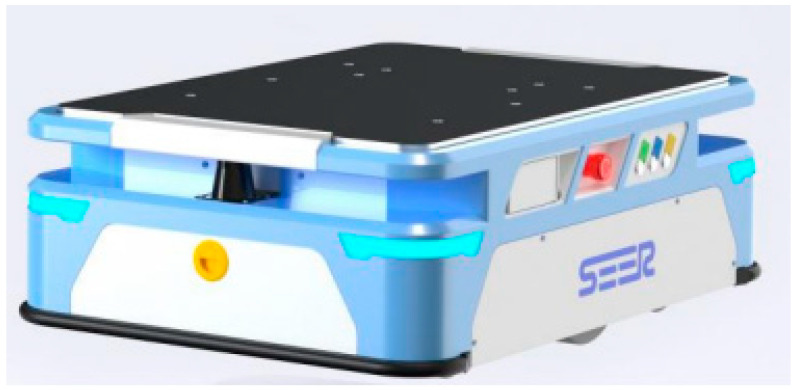
AGV from SEER, type AMB-J [33].

The first alternative option we chose was a jacking transfer robot from SEER, specifically the AMB-J type (see Figure 4). The robot uses a two-wheel differential drive mode. The maximum speed achieved with the load is 1.5 m/s. The device is a manipulator robot with a jacking mechanism, which is based on an operation using simultaneous localization and mapping (SLAM) lasers [33]. SLAM is the process of mapping an area while tracking the location of a device in that area. This is what mobile mapping enables. This allows large areas to be mapped in much less time because areas can be surveyed using mobile robots, drones, or vehicles. SLAM systems simplify data collection and can be used in outdoor or indoor environments [34].

The AMB-J types are latent jack-up robots, with payloads covering 150, 300, and 600 kg. The unit has compact dimensions: it is 780 mm long, 550 mm wide, 260 mm high, and weighs 135 kg. The maximum lift height is 50 mm with tolerances of ±2 mm and the turning diameter is 910 mm. Due to its small size, the vehicle is very flexible and can fit into very narrow streets. Its low height makes it suitable for low-lying trolleys or shelves and racks. The automated guided vehicle can identify different shelves, racks, and other components in the warehouse. Multiple security is provided by staggered dual lasers located on both the front and back. Fast charging has a significant positive impact on the battery life, enabling it to operate continuously for a full 24 h. The lithium battery can be charged in auto-charging mode or manually and has a duration of 8 h on a full charge. A continuous connection of 3 h is required to fully charge the battery through either continuous charging or being connected to the charger [33].

The selected AGV model also has specified conditions when it cannot be deployed and used. The prohibitions of operation are as follows:

- Must not be used outdoors;
- Must not be used in an environment that would severely interfere with the navigation equipment;
- Should not be used in environments with dust, powder, or explosive hazards;
- Is not allowed to be used in highly salted environments (marine climate);
- Must not be used in extremely harsh weather (cold, strong magnetic fields and extreme weather);
- In terms of materials, the carriage of explosive and flammable materials is prohibited, and these materials, liquid objects and persons are not allowed to be transported on it;
- From the perspective of the terrain, riding on uneven, obstructive, or stepped surfaces is prohibited [33].

The software offers the following features: editing of maps and models, a module for localization, a module for navigation, peripheral extension, an application programming interface (API), visible operation, the dispatching of multiple robots, Wi-Fi, automatic charging, laser reflector navigation, and pallet recognition [33].

#### 3.2.2. Navigation and Control System

This system enables the robot to carry out a range of predetermined tasks. Within the created map, the navigation and control system directs the robot to a specified destination, as instructed by the user. The procedure consists of:

- Global path planning—this involves identifying the destination point and inputting a map file to generate the shortest route to the destination. It also includes breaking down one large task into sub-tasks for each route section;
- Task scheduling—based on the subtasks of the current route or action execution module, the task scheduler generates auxiliary tasks according to the specified instructions;
- Local path planning—the robot only advises movement along the current specified route. If an obstacle occurs on its route, based on the current position of the route given by the localization device and using sensors, the system will block, report an error, and then lock (stay in place);
- Action planning—the robot is set to perform the specified action based on instructions and current information from the sensors;
- Localization—the robot has a built-in localization module that provides real-time accurate information about its location, based on SLAM navigation, and also uses navigation using QR codes;
- Sensors—used to detect obstacles and possible assessments for slowing down, there are built-in sensors such as a six-axis gyroscope, sensor, 3D camera, and infrared and DI signal, etc.;
- Actuators—these are used to measure the state of the robot and, when executed following a stimulus, execute the specified tasks [33].

When introducing AGVs, it is not enough to acquire the robot itself, but also the accessories necessary for its proper functioning, which are as follows: the charging station and the corresponding charging cables, the safety module, software, and training, etc. And the cost also includes all the transport of the robot to the company, packaging, installation, and wiring costs.

#### 3.2.3. Proposal to Introduce AGV/AMR in the Warehouse

When AMR/AGV is proposed to be introduced into the company, only one such vehicle is required, which reduces the number of current AGVs (2). The implementation of AMR/AGV has been specified only for AGVs that are small and have the loads positioned on top of each other. A technology using AGV/AMR vehicles would follow the same principle of material transport, with the vehicle being equipped with a lifting platform. The introduction of 1 vehicle would also minimize the number of vehicles in the handling aisle, which has the impact of reducing congestion.

**Figure 5:**
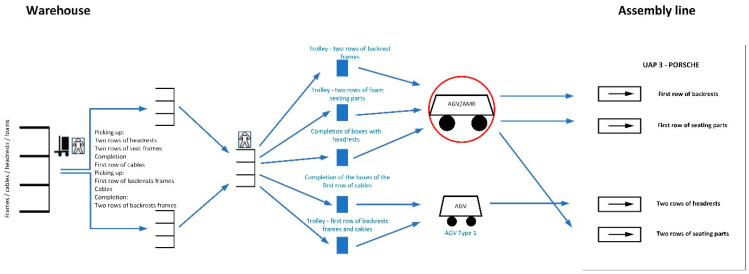
Scheme for the introduction of AMR/AGV vehicles.

The AGV/AMR deployment can be seen in the diagram above (Figure 5), in which the AGV/AMR is indicated in the red circle.

#### 3.2.4. Time Use

Table 3 displays the production volume of seats designated for the chosen vehicles, denoted by the letter “C” in the table. The table also outlines the distribution of seats by comfort type such as classic, comfort, or sport. The production hall has a three-shift operation with a current output of 510 seats per day, equivalent to 170 seats per shift.

One working shift, i.e., 8 h (480 min), is distributed as follows: 1st break—10 min, 2nd break—20 min; 5 min at the beginning of the shift, where the authorized worker explains any changes to the operators, and 5 min at the end of the shift, which belongs to the cleaning of the hall, are still included in the rest time. This gives a networking time of 440 min per shift.

#### 3.2.5. Before the Introduction of AGVs

The production line requires 28 auto-sets of each material per hour. As a result, the transport routes for a specific cycle were adjusted accordingly. Table 4 presents the number of car-sets transported per transport trolley, with distances in meters. The distance from the warehouse to the production line is the same when transporting the same type of material. Table 4 presents additional details, such as the hourly demand for trolleys, the operator’s theoretical speed, and the total cycle time, which includes the time taken to stop work and retrieve an empty trolley, as well as a 50% congestion efficiency value. This calculation includes the previous period’s AGVs, which required the trolley operator to give way before crossing the handling aisle, resulting in downtime. As a result, the three workers who were responsible for supplying materials were also responsible for loading the trolley in the material warehouse. This time is no longer considered as part of the transport cycle.

#### 3.2.6. After the Introduction of AGVs

The information needed to calculate the cycle time is again shown in Table 5, where the distance information has not changed with respect to the previous technology. Next, the table shows the measured values exhibited by the AGV. These mainly relate to the material required in the line, which has remained unchanged because the company has signed contracts for a certain period that it has to fulfil, and for this reason the requirement has remained unchanged. The subsequent supply of the assembly line depends on this parameter. Subsequently, there is information on the quantity of material transported; where the capacity of the trolleys has been doubled for the foam reinforcements and for the head restraints this has increased in the number of pieces, from 12 pieces to 45 pieces. As a result, there is no need for such an intensive amount of transport of the picked-up trolleys to the line, as with the previous technology. This also reduces the density in the handling line. Another parameter is the AGV speed, which is almost twice as low as that of the operator, which also affects the total cycle time. Not least, we can also note the number of stops in the loop, which reaches 4. This sequence of stops is as follows: first, the cart is loaded in the warehouse near the AGV. Second, the cart is unloaded on the assembly line. Third, the empty cart is picked up in the line area. Lastly, the empty cart is unloaded near the warehouse. Each single stop represents a time of 30 s. The AGV stops also need to be included in the cycle-time calculation, due to congestion that occurs in the handling aisle. The coefficient is given by the company and is 80%; also, the additional item of time lost due to accelerations and decelerations of the AGVs is also 80%.

#### 3.2.7. Time Payback on Investment

Time payback is a crucial metric for companies. It is particularly useful when assessing potential investments in innovative ventures, where calculating the time taken to recuperate an initial spend is essential. For a company, this is one of the main elements that has sufficient influence on the decision of the suitability of an investment. From this, we can understand the net profit in each period. At the beginning of the period, the values show a net loss, and the consequence of these results is the finance invested in innovation. It is up to the company which method it uses to calculate the return, as the difference in methods is the range of items included. The most used method in companies is the share of net profit minus the initial investment method; it is also possible to express it as a percentage. In terms of accuracy, the method is the so-called mean payback period, which is much more accurate than the above method. In this method, the calculation is as follows: the cost of the investment is divided by the annual cash flow (annual income) minus the cost savings of the investment.

The general formula for return on investment (*ROI*) is as follows:

(1) $$ROI=\;C_{ap}-C_{amr}$$

where 

*ROI*—cost of implementation (e.g., technology) [EUR/month];

*C_ap_*—average past costs [EUR/month];

*C_amr_*—average maintenance and repair costs [EUR/month] [7].

The cost of electricity is not required by the company, so we will only count it on input data. We will also not count the cost of carrying out repairs and maintenance work because the company, almost since the introduction of the first automation, has a special in-house employee who is responsible for carrying out repairs and maintenance.

#### 3.2.8. Costs Incurred from the Previous Technology

This section summaries the costs of the previous technology used by the company before the introduction of the AGVs. Cost items include the wages of the employees in each segment. In the problem addressed, there were three employees who removed the material and then transported it from the warehouse to the assembly line. They also transported empty carts from the assembly line to the warehouse. This is the cost per employee (see Table 6). The table also shows the total cost in EUR per month.

When using human labour, the highest cost to society is the wage paid to employees. As the cost of human labour rises every year, rising in direct proportion to inflation, so do the costs to society.

#### 3.2.9. AGV Investment in the Company

The company provided us with real data on the total costs of the introduction of the AGV technology. The total cost will be found for the items that include the purchase of the AGV itself, and there are also the items related to the proper functioning of the AGV, such as installation, putting the AGV into service, transport, project design and others. In addition, we included in the cost of technology the carts, which were newly designed to fit the AGV. Table 7 shows the additional costs for infrastructure modification and hall adaptation. New guidance elements are also included in the cost. The company handled the acquisition of AGV in two ways.

The first method of financing was carried out by monthly instalments; in this way, the accessories for the operation of the AVG were procured, which the company had to purchase. The second method was also financed by monthly instalments, where the company does not purchase the AGVs but only leases them. The monthly instalment of the AGV rental is EUR 450.

Table 8 shows the calculation of the returns between the previous and the current technology introduced to the firm on Segment 2. From the table, we can see the next total annual cost of the previous technology is EUR 79,200 and the total cost associated with the current technology (AGV) is EUR 49,000.

By implementing AGVs in Segment 2, a time payback compared to the previous cost per worker, in a time period of 8 months, was created.

## 4. Conclusions

By implementing two AGVs, we were able to replace the human work of four operators. It was a constantly repeating handling route consisting of four stations using different modes of transport. Based on the workload analysis of the individual employees, we concluded that this was a suitable place to implement AGVs for the positions because of the repeatability and the one-sidedness of the individual operations. 

Financially, the company saves EUR 24,824 per year, due to the replacement of old AGVs with new AGVs in the service. Up until now, 18 operators have been needed for individual operations, whose annual wage costs amount to EUR 28,000. The cost of operating flood equipment such as low-lift trucks, forklifts and small trains amounted to EUR 61,000 per year in the company. Since the introduction of the first AGV, the company has saved EUR 756,000 in wage costs for 18 people. The resulting calculation for the company’s position, which we have studied, shows that within 8 months the company will have recovered the financial resources invested in AGVs. Another but more financially demanding option would be the replacement of AGVs with AMRs, which we have tried to point out, and which can also be identified as very realistic in terms of cost recovery. The only question is which way the company will choose in terms of technology. The cost calculations for the given technologies and their advantages and disadvantages are described in this article. The company will continue to automate the processes, because it increases safety in the company and the degree of comfort, speed, and simplicity of the equipment of individual performances. 

By implementing AGVs in their own way, the company acquired two automated vehicles with a return on investment of 8 months. The company’s investment in the AGVs amounts to EUR 79,200 for the selected model, saving the work of three employees who can be reassigned to operations that the AGV cannot perform.

The company is constantly striving to introduce automated vehicles with positive results in terms of savings and forms of the automation process.

In our case, we had all the information we needed to perform a detailed analysis. The necessary information consisted mainly of the technical parameters of the vehicles and trolleys, the price, uptime, and line utilization, and finally, the calculated parameters of both time and economic payback.

## Figures and Tables

**Table 1:** The remaining parameters of the AGV.

Parameter	Reported Value
Max. load on top surface with towing trolley	450 kg 1000 kg
Max. speed with or without load	2.7 km/h
Width, Length, Height	800 mm, 800 mm, 280 mm
AGV weight	215 kg

**Table 2:** Summary of the logistics efficiency information.

Cycle time of the Manufactoring [s]	155
Working time in minutes to complete goods	31.8
Ideal number of operators	12.27
Planned number of operators	44
Productivity potential (%)	72%
Planned logistics time [minutes/end of product]	113.9
Current number of shift operators	43
Efficiency of direct work in logistics	29%

**Table 3:** Summary of production volume.

Segment 2							Total
Type of Seat	Classic	Comfortable	Sports	C8 Way	C Comfort	C Sport	
Production volume [%]	6	60	7	6	13	8	100
Volume/day [pcs]	31	304	36	33	68	38	510
Number of shifts/days	3	3	3	3	3	3	-
Volume/shift [pcs]	10	101	12	11	23	13	170

**Table 4:** Time use of operators (4.2).

Ordinal Number	Material Transported	Required Quantity/in Line/h [pcs]	Quantity of Car-Sets/Trolley	Name of the Flow	Pickup	Unloading	Distance There (m)	Distance Back (m)	Quantity of Trolleys	Quantity of Trolleys/h	Theoretical Speed (m/s)	Time/Stop (s)	Congestion Efficiency (%)	Transport Time/Cycle (s)
1.	Two rows of foam seating	28	6	Flow 1	P1	D1	45	45	1	4.7	1.1	4	50	183
2.	Two rows of foam backrests	28	6	Flow 2	P2	D2	40	40	1	4.7	1.1	4	50	169
3.	Two rows of headrests and backrests	28	4	Flow 3	P3	D3	55	55	1	7	1.1	4	50	210

**Table 5:** Time use of AGVs.

Ordinal number	Material Transported	Required Quantity/in Line/h [pcs]	Quantity of Car-Sets/Trolley	Name of the Flow	Pickup	Unloading	Distance There (m)	Distance Back (m)	Quantity of Trolleys	Quantity of Trolleys/h	Theoretical Speed (m/s)	Time/Stop (s)	Congestion Efficiency (%)	Time Loss Due to Turning or Deceleration (%)	Transport Time/Cycle (s)	Practical Number of AGV/Loop (pcs)
1.	Two rows of foam seating	28	10	Flow 1	P1	D1	45	45	1	2.8	0.4	4	30	80	539	2
2.	Two rows of foam backrests	28	12	Flow 2	P2	D2	40	40	1	2.3	0.4	4	30	80	500	
3.	Two rows of headrests and backrests	28	15	Flow 3	P3	D3	55	55	1	1.9	0.4	4	30	80	617	

**Table 6:** Employee costs.

Activity Performed	Number of Operators	Total Unit Cost/Month (EUR)	Total Unit Cost/Year (EUR)
Warehouse operator	1	2200	26,400
Trolley operator	2	4000	52,800
**Total costs (EUR)**		**7000**	**79,200**

**Table 7:** Summary of AGV costs in the company.

Item	Quantity (pcs)	Unit Price (EUR)	Total Price (EUR)
**AGV costs**			**70,550.00**
AGVs and accessories	2	18,500	37,000
Lease finance costs (excluding VAT)	48	1350	27,800
Insurance (small recurring costs)	48	-	-
Project	1	5000	5000
Transport	1	750	750
Installation	1	-	-
Commissioning (performed by the AGV team)	0	-	-
Spare parts	1	-	-
**Trolleys**			**21,700**
Trolleys	5	1500	7500
Renewal of stands	2	2000	4000
Training	1	8000	8000
Supplementary material for trolleys	4	300	1200
Sensors, cables, light indication for trolley	2	500	1000
**Warehouse modification**			**10,750**
Preparation of trolley loading points	1	8000	8000
Preparation of trolley unloading points	0	3000	-
Tape—layout reorganization	400	5	2000
Tags and protection	100	8	750
**Total cost of AGV 1**			**38,200**
**Total cost of AGV 2 (lease)**			**64,800**

**Table 8:** Summary of costs and investments.

Calculation of Return	Quantity (pcs)	Unit Price (EUR)	Total Price (EUR)
Large one-off costs	-	-	38,200
Small additional costs:			
AGV rental costs (450 EUR/month)	24	450	10,800
Maintenance	0	100	-
**Total cost**			**49,000**
Staff	3	26,400 EUR/year	79,200 EUR/year

Time returns on finance AGV ROI = 49,000/79,200 = 0.6187 = 0.6187 years = 8 months.

## Data Availability

Data are contained within the article.

## References

[1] Xie L., Li H., Luttmann L. (2023). Formulating and solving integrated order batching and routing in multi-depot AGV-assisted mixed-shelves warehouses. Eur. J. Oper. Res..

[2] Qin H., Xiao J., Ge D., Xin L., Gao J., He S., Hu H., Carlsson J.G. (2022). JD.com: Operations Research Algorithms Drive Intelligent Warehouse Robots to Work. Inf. J. Appl. Anal..

[3] Jang J.-Y., Yoon S.-J., Lin C.-H. (2023). Automated Guided Vehicle (AGV) Driving System Using Vision Sensor and Color Code. Electronics.

[4] Kaven L., Abele E., Göppert A., Schmitt R.H. (2022). Digital Twin Pipeline for VDA 5050 Integration A Standardised Approach for the Automated Implementation of Flexible Production. ZWF Z. Fuer Wirtsch. Fabr..

[5] Wu S., Xiang W., Li W., Chen L., Wu C. (2023). Dynamic Scheduling and Optimization of AGV in Factory Logistics Systems Based on Digital Twin. J. Appl. Sci..

[6] Reis W.P.N.d., Couto G.E., Junior O.M. (2023). Automated guided vehicles position control: A systematic literature review. J. Intell. Manuf..

[7] Cheong H.-W., Lee H. (2018). Requirements of AGV (Automated Guided Vehicle) for SMEs (Small and Medium-sized Enterprises). Procedia Comput. Sci..

[8] Nagayoshi M., Elderton S.J.H., Sakakibara K., Tamaki H. (2017). Reinforcement learning approach for adaptive negotiation-rules acquisition in AGV transportation system. J. Adv. Comput. Intell. Intell. Inform..

[9] Sun M., Lu L., Ni H., Wang Y., Gao J. (2022). Research on dynamic path planning method of moving single target based on visual AGV. SN Appl. Sci..

[10] Ondrej S. (2020). Modeling the Delivery Routes Carried out by Automated Guided Vehicles when Using the Specific Mathematical Optimization Method. Open Eng..

[11] Mantel R., Landeweerd H. (1995). Design and operational control of an AGV system. Int. J. Prod. Econ..

[12] Lu S.P., Xu C., Zhong R.Y., Wang L.H. (2017). A RFID-enabled positioning system in automated guided vehicle for smart factories. J. Manuf. Syst..

[13] Sun S., Hu J., Li J., Liu R., Shu M., Yang Y. (2019). An INS-UWB Based Collision Avoidance System for AGV. Algorithms.

[14] Manikandan S., Kaliyaperumal G., Hakak S., Gadekallu T.R. (2022). Curve-Aware Model Predictive Control (C-MPC) Trajectory Tracking for Automated Guided Vehicle (AGV) over On-Road, In-Door, and Agricultural-Land. Sustainability.

[15] Schweitzer F., Bitsch G., Louw L. (2023). Choosing Solution Strategies for Scheduling Automated Guided Vehicles in Production Using Machine Learning. Appl. Sci. Open Access.

[16] Iza B.A., Fiddina Q.A., Fadhilah H.N., Arif D.K., Mardlijah M. (2022). Automatic Guided Vehicle (AGV) tracking model estimation with Ensemble Kalman Filter. AIP Conference Proceedings.

[17] Hu Y., Yang H., Huang Y. (2022). Conflict-free scheduling of large-scale multiload AGVs in material transportation network. Transp. Res. Part. E Logist. Transp. Rev..

[18] Bernardo R., Sousa J.M.C., Gonçalves P.J.S. (2022). Survey on robotic systems for internal logistics. J. Manuf. Syst..

[19] Clauer D., Fottner J., Rauch E., Prüglmeier M. (2021). Usage of Autonomous Mobile Robots Outdoors—An Axiomatic Design Approach. Procedia CIRP.

[20] Aliev K., Traini E., Asranov M., Awouda A., Chiabert P. (2021). Prediction and estimation model of energy demand of the AMR with robot for the designed path in automated logistics systems. Procedia CIRP.

[21] Loganathan A., Ahmad N.S. (2023). A systematic review on recent advances in autonomous mobile robot navigation. Eng. Sci. Technol. Int. J..

[22] Zhang J., Yang X., Wang W., Guan J., Ding L., Lee V.C. (2023). Automated guided vehicles and autonomous mobile robots for recognition and tracking in civil engineering. Autom. Constr..

[23] Popova I., Abdullina E., Danilov I., Marusin A., Ruchkina I., Shemyakin A. (2021). Application of the RFID technology in logistics. Transp. Res. Procedia.

[24] Yue G., Tailai G., Dan W. (2021). Multi-layered coding-based study on optimization algorithms for automobile production logistics scheduling. Technol. Forecast. Soc. Chang..

[25] Costa A.F., Carvalho M.D.S., Henriques M., Ferreira P.V. (2022). Strategy for the introduction of autonomous driving technologies: A case study in the logistics area of an automotive company. Procedia Comput. Sci..

[26] Bohács G., Győrváry Z., Gáspár D. (2021). Integrating scheduling and energy efficiency aspects in production logistic using AGV systems. IFACPapersOnLine..

[27] Maoudj A., Kouider A., Christensen A.L. (2023). The capacitated multi-AGV scheduling problem with conflicting products: Model and a decentralized multiagent approach. Robot. Comput. -Integr. Manuf..

[28] Vlachos I.P., Pascazzi R.M., Zobolas G., Repoussis P., Giannakis M. (2023). Lean manufacturing systems in the area of Industry 4.0: A lean automation plan of AGVs/IoT integration. Prod. Plan. Control.

[29] Steclik T., Cupek R., Drewniak M. (2022). Automatic grouping of production data in Industry 4.0: The use case of internal logistics systems based on Automated Guided Vehicles. J. Comput. Sci..

[30] Kengpol A., Elfvengren K. (2022). Avoiding Covid-19 Using a 3D Digital Mock up and Augmented Reality with Cobot in Digital Factory. Appl. Sci. Eng. Prog..

[31] van Gils T., Ramaekers K., Caris A., de Koster R.B.M. (2018). Designing efficient order picking systems by combining planning problems: State-of-the-art classification and review. Eur. J. Oper. Res..

[32] Kostrzewski M. (2020). Sensitivity Analysis of Selected Parameters in the Order Picking Process Simulation Model, with Randomly Generated Orders. Entropy.

[33] Li J., Cheng W., Lai K.K., Ram B. (2022). Multi-AGV Flexible Manufacturing Cell Scheduling Considering Charging. Mathematics.

[34] Thylén N., Wanstrom C., Hanson R. (2023). Challenges in introducing automated guided vehicles in a production facility—Interactions between human, technology, and organization. Int. J. Prod. Res..

